# Functional and radiological changes in ICU survivors with COVID-19 at 1-year follow-up from the OUTSTRIP COVID-19 study

**DOI:** 10.5339/qmj.2025.40

**Published:** 2025-06-30

**Authors:** Merlin Thomas, Mousa Hussein, Amal Al Rashid, Maysa Mohamed, Aalaa Kambal, Rajendra Kumar, Mansoor Ali Hameed, Rajvir Singh, Mushtaq Ahmad, Saibu George, Jaweria Akram, Aisha Hussain O. Al Adab, Rajalekshmi Maheswari Rajagopal, Rohit Sharma, Tasleem Raza

**Affiliations:** 1 Department of Chest, Hamad General Hospital, Doha, Qatar; 2 Department of Clinical Medicine, Weill Cornell Medicine-Qatar, Ar-Rayyan, Qatar; 3 Department of Clinical Medicine, Qatar University, Doha, Qatar; 4 Department of Radiology, Hamad General Hospital, Doha, Qatar; 5 Medical Research Centre, Hamad General Hospital, Doha, Qatar; 6 Department of Medical Intensive Care, Hamad General Hospital, Doha, Qatar; 7 Hamad General Hospital, Doha, Qatar; 8 Department of Internal Medicine, Geisinger Health System, Danville, PA, USA *Email: mmts1983@gmail.com

**Keywords:** COVID-19, radiology, CT chest, lung function, exercise capacity, ICU survivors

## Abstract

**Objective:**

We aimed to determine the radiological changes on computed tomography (CT) scans of the chest and pulmonary function abnormalities at a 1-year follow-up in intensive care unit (ICU) survivors with severe COVID-19.

**Design:**

A 2-year prospective cohort study with an enrollment of 204 patients within 3 months after their discharge from the ICU at Hamad General Hospital, the tertiary care center in Qatar.

**Main Results:**

The mean age of our cohort was 48.7 ± 8.6 years. CT chest was performed on a total of 135 patients at a 1-year follow-up, out of which 43 patients had a CT chest during hospital admission with COVID-19. Abnormal CT chest findings were seen in 118 (87.4%) patients at 1 year. The mean CT severity score significantly improved at 1 year (8.1 ± 6.8 vs. 19.4 ± 3.6, *p* < 0.001). Those with an abnormal CT chest in 1 year had a significantly lower but normal predicted forced vital capacity (91.9% ± 15.4 vs. 81.1% ± 13.7, *p* = 0.01), predicted total lung capacity (82.5% ± 13.9 vs. 94.3% ± 12.7, *p* = 0.02) and oxygen saturation after 6-minute walk test (6MWT) (97.5 ± 1.2 vs. 98.3 ± 0.651, *p* = 0.033). 6MWT distance was significantly shorter than the predicted distance in those with an abnormal CT (63 (62.4%) vs. 7 (77.8%), *p* = 0.020).

**Conclusion:**

Patients recovering from severe COVID-19 have significant improvement but persistent radiological changes at a 1-year follow-up that correlate with several physiological parameters, but these findings are limited by the absence of pre-COVID-19 baseline imaging.

## Key points

- **Question:** What are the long-term pulmonary radiological and physiological outcomes at 1-year follow-up in ICU survivors with COVID-19?
- **Findings:** This prospective cohort study showed that 87.4% of patients have abnormal CT chest in 1 year with significant improvement in CTSS (8.1 ± 6.8 vs. 19.4 ± 3.6, *p* < 0.001) and demonstrate a shorter predicted walk distance on 6MWT (63 (62.4%) vs. 7 (77.8%), *p* = 0.020).
- **Meaning:** A significant proportion of ICU survivors of COVID-19 exhibit one or more abnormal chest CT patterns at 1 year with impairment in 6-minute walk distance.

## INTRODUCTION

During the initial phase of the COVID-19 pandemic, a significant proportion of patients experienced severe illness necessitating hospitalization, with nearly 20% falling into this category. Of those hospitalized, a quarter required admission to the intensive care unit (ICU).^1,2^ As of March 2023, Qatar has reported approximately 500,000 COVID-19 cases, with less than 4 out of every 1000 cases being severe or critical enough to warrant ICU admission and hence reported one of the lowest mortality rates.^3,4^ These rates of severe infection requiring ICU care stand in stark contrast to the figures reported in many other countries, underscoring the influence of various contributing factors.^4^

The course of acute COVID-19 disease exhibits considerable variability, ranging from asymptomatic cases to severe respiratory failure. Recovery for patients is further complicated by the persistence of symptoms, pulmonary physiological impairments, and observable radiological changes. The etiology of these enduring alterations can be attributed to factors such as direct viral injury, the aftermath of acute respiratory distress syndrome (ARDS), and lung injuries resulting from mechanical ventilation.^5^ Numerous publications have detailed short-, medium-, and long-term outcomes for survivors of ICU care with varying degrees of COVID-19 severity.^6–9^ Radiological changes tend to improve over time, but a significant portion persists, leading to residual lung fibrosis that is largely nonprogressive and several other risk factors have been associated with these findings.^10^ In the acute phase of COVID-19, autopsy studies of mechanically ventilated patients revealed significant hyperplastic and metaplastic changes in pneumocytes, as well as prominent interstitial fibrosis, compared to patients receiving conventional oxygen therapy. Similarly, early HRCT findings during hospitalization have shown fibrotic changes, suggesting that SARS-CoV-2 may directly injure pulmonary alveolar and endothelial cells. This injury, combined with an abnormal local immune response, could increase the risk of pulmonary fibrosis in susceptible individuals.^11,12^

Studies investigating radiological outcomes at the 1-year mark are relatively limited in scope, often comprising small patient cohorts (fewer than 100 individuals) and encompassing heterogeneous populations with varying degrees of disease severity. Long-term outcome data, particularly from Gulf countries with unique population characteristics, remains scarce. Qatar boasts a diverse population characterized by a median age of 32.3 years, with non-Qatari residents constituting a majority (88.4%) of the population.^13^ Qatar’s relatively young and predominantly expatriate population, coupled with its centralized, government-funded healthcare system that allows access to advanced medical care, including ICU facilities and standardized follow-up, which likely facilitated early diagnosis, consistent management, and comprehensive follow-up for patients, may have influenced the long-term recovery and mortality patterns in Qatar.

This paper reports on the findings of the OUTSTRIP COVID-19 study, a 2-year prospective investigation conducted at Hamad Medical Corporation (HMC) in Qatar.^14^ Our primary objective was to examine the long-term outcomes in adult patients with severe COVID-19 within Qatar. In this paper, we present our findings regarding pulmonary radiological changes and physiological outcomes, as measured by pulmonary function tests (PFTs) and the 6-minute walk test (6MWT), 1 year following discharge from the ICU.

## METHODS

The OUTSTRIP COVID-19 study was conducted at HMC, a tertiary care center in Qatar. Regulatory and ethical approval was granted by the Medical Research Centre of HMC under research protocol number MRC-05-073.

## STUDY DESIGN AND PARTICIPANTS

The inclusion and exclusion criteria, as well as the case definition for severe/critical COVID-19, were previously published in a paper by the same study group^14^ and included patients ≥18 years with SARS-CoV-2 confirmed by RT-PCR, admitted to the ICU for severe/critical COVID-19, and able to provide informed consent before discharge or within 7 weeks at COVID chest clinics. Exclusion criteria included suspected acute brain lesions (e.g., stroke, traumatic brain injury, hypoxic brain injury), preexisting neuropsychological conditions, moderate-to-severe chronic obstructive pulmonary disease (COPD), asthma, cystic fibrosis, or parenchymal lung disease.^11^ Pertinent to this manuscript, patients known to have the following conditions prior to COVID-19 were excluded from the study: moderate-to-severe COPD, bronchial asthma, cystic fibrosis, and parenchymal lung diseases, including interstitial lung disease. We enrolled a total of 204 patients into the study within 3 months after their discharge from the ICU. After 1 year, we conducted computed tomography (CT) scans of the chest, PFTs, and 6MWTs on 135 of these patients. Participant dropout occurred randomly rather than systematically. Among these 135 patients, 43 had previously undergone a CT chest scan during their hospital admission for COVID-19 as part of their clinical care.

## DATA COLLECTION

### CT protocol and image analysis

The CT examination at the 1-year mark was conducted using a Siemens SOMATOM Definition Drive/Edge/AS/Flash multidetector CT scanner with 128 slices; dual/single source; dual energy with 70 kV/140 kV; Software/ADMIRE. The specific parameters for CT acquisition included a tube voltage of 120 kVp, standard tube current (with a reference mAs range of 60–120), a slice thickness of 1.0 mm, and a reconstruction interval of 1.0 mm. All CT images were obtained with the patients in a supine position during full inspiration and without the use of a contrast medium, utilizing a low-dose technique. For image evaluation, CT images were viewed on PACS workstations, employing a width of 1500 HU (Hounsfield units) and a level of −600 HU for the pulmonary window, as well as a width of 350 HU and a level of 50 HU for the mediastinal window. Two pairs of consultant radiologists, each with more than 5 years of experience in thoracic radiology, independently reviewed all CT images at baseline and 1-year follow-up. These radiologists were blinded to the patients’ baseline clinical characteristics and outcomes. In cases of disagreements, consensus was reached through discussion, and the final report was analyzed.

The CT assessment focused on the following characteristics: the distribution of abnormalities (whether peripheral, central, or a combination of both, or neither peripheral nor central), the affected lung lobes (right upper, middle, and lower lobes, and left upper and lower lobes), and the number of lobes involved (ranging from one to five). Following the Fleischer Society glossary,^15^ predominant CT patterns were categorized, encompassing ground-glass opacity, consolidation, the presence of nodules or masses, “crazy-paving” patterns, air bronchograms, emphysema, reticulation, honeycombing, bronchiectasis, architectural distortion, fibrous stripes, subpleural lines, and vascular thickening. Extrapulmonary manifestations such as enlarged mediastinal lymph nodes, pleural thickening, and pleural effusion were also documented. Furthermore, the presence of traction bronchiectasis, parenchymal bands, and/or honeycombing indicated fibrotic-like changes.^16^ Based on the extent of involvement of the five lung lobes, a semiquantitative CT score was assigned to quantify the abnormalities. This score was categorized as follows: 0 (no involvement), 1 (involvement of less than 25%), 2 (involvement ranging from 25% to 50%), 3 (involvement between 51% and 75%), and 4 (involvement exceeding 75%). The summation of these individual lobar scores produced a total CT score ranging from 0 to 25.^17^

## PULMONARY FUNCTION TEST AND 6-MINUTE WALK TEST

The PFT and the 6MWT were conducted at our Lung Function Laboratory, accredited by the American Thoracic Society (ATS). PFT was administered using a body plethysmograph from NDD, © ndd Medizintechnik AG, CH 8005 Zurich, following ATS guidelines.^18^ This encompassed the measurement of forced vital capacity (FVC), forced expiratory volume in 1 second (FEV1), diffusion capacity of lungs for carbon monoxide (D_LCO_), and alveolar ventilation (V_A_), all expressed as percentages of predicted values as per the Global Lung Function Initiative 2012 standards.^19^ Additionally, residual volume (RV), FEV1/FVC, D_LCO_/V_A_, and D_LCO_ adjusted for hemoglobin were calculated. We determined the number of participants meeting commonly used clinically relevant cutoffs for FVC (<80% of predicted), FEV1 (<80% of predicted), FEV1/FVC (<70% of predicted), D_LCO_ (<70% of predicted), and O_2_ saturation (<92%).^20^

The 6MWT was conducted in accordance with accepted standards and protocols.^20,21^ At the outset and conclusion of the 6MWT, we recorded baseline pulse, oxygen saturation measured using the Nonin Model 7500 pulse oximeter, and blood pressure and utilized the modified Borg scale to assess dyspnea and fatigue in all patients. During the test, participants walked at their own pace along a 30-m hallway. Reference values were calculated employing Enright’s equation for healthy adults.^20^ We also reported heart rate recovery (HRR) by assessing the pulse rate after 1 minute of rest following the 6MWT, with a relevant cutoff set at 13 beats/minute.^23^

## STATISTICAL ANALYSIS

To summarize the quantitative data, we calculated the mean and standard deviation (SD) for interval variables, and for categorical variables, we presented frequencies and percentages. Lung function and 6-minute walk parameters were described. Data normality was assessed using the Kolmogorov-Smirnov test before applying the appropriate statistical tool for interval variables. Demographic, clinical, biochemical, oxygenation—ventilation and prognostic characteristics with CT chest at 1 year were compared between normal CT vs. abnormal CT. For comparisons between the two groups, we utilized independent t-tests or Mann-Whitney U tests as appropriate for interval variables. For categorical variables, we employed chi-square tests (or Fisher’s exact tests when necessary). Similar tests were applied for chest features during admission with COVID-19 and at a 1-year follow-up between patients in 1 year and patients at baseline and associations of abnormal CT chest findings at follow-up with pulmonary outcomes. A significance level of 0.05 (two-tailed) was considered statistically significant. We performed the analysis using the SPSS version 29.0 statistical software package.

## RESULTS

Table 1 presents the baseline clinical characteristics of our cohort. The patient group consisted predominantly of males (78%), with an average age of 49 years. Notably, diabetes mellitus and hypertension emerged as the dominant comorbidities.

In Table 2, we detail the baseline hematological and ventilation parameters. Upon admission to the ICU, the mean lymphocyte counts exceeded 1 × 10^3^/μL, accompanied by a mean C-reactive protein level of 150 mg/L. Additionally, Ferritin levels averaged 2600 μg/L, and D-dimer levels were recorded at 6.1 mg/L.

Concerning oxygenation, most patients (91.5%) exhibited critical respiratory failure, as evidenced by the low PaO_2_/FiO_2_ ratio. Moreover, the APACHE II score at the 24-hour mark fell within the range of 5–9 for 56% of the patients.

It is worth noting that all patients necessitated respiratory support, with 74.6% receiving high-flow nasal cannula (HFNC), 71.9% utilizing noninvasive ventilation (NIV) through either Bilevel positive airway pressure or continuous positive airway pressure, while only 23.7% required invasive mechanical ventilation (IMV). The mean length of ICU stay was 10.8 days.

## ANALYSIS OF RADIOLOGICAL FINDINGS ON CT CHEST

During admission for COVID-19, all patients who underwent a CT chest showed abnormal findings, in contrast to 118 (87%) patients who still exhibited abnormal CT chest results at 1-year follow-up. Notably, the mean CT severity score (CTSS) significantly decreased at the 1-year follow-up compared to the baseline measurements, registering at 8.1 ± 6.8 vs. 19.4 ± 3.6 (*p* = 0.001) (Table 3).

At baseline, the most prevalent abnormality was a mixed pattern of ground-glass opacities (GGO) combined with consolidation, observed in 30 (69.8%) patients. Conversely, at the 1-year follow-up, reticular changes were evident in 90 (66.7%) patients. Fibrotic changes were significantly more common at the 1-year follow-up compared to baseline (68% vs. 23.2%, *p* = 0.001). Among these, the majority exhibited a mixed pattern, featuring subpleural lines, fibrous cord shadows, and traction bronchiectasis.

## RELATIONSHIP BETWEEN PFT, 6MWT, AND RADIOLOGY

Only 47% of the 99 patients who underwent a PFT at the 1-year mark exhibited abnormalities, with the predominant finding being a restrictive defect observed in 45% of these patients. The mean values for all lung function parameters (Table 4) fell within normal limits. Among the patients who performed PFT, 87 had an abnormal CT chest scan. Among this subgroup with an abnormal CT chest, 42 (42.4%) displayed normal PFT results, while 2 (2.0%) showed obstructive and 43 (43.4%) had a restrictive pattern. Within this group of patients with restriction, 32 (32.3%) had a total lung capacity (TLC) less than 80% of the predicted value.

When assessing functional capacity through the 6MWT (Table 4), 62% of the patients covered distances shorter than the predicted values, and 19(18.8%) experienced a decrease in oxygen saturation by 4% or more at the end of the test. Within the subgroup displaying significant end-of-test desaturation, 6(31.5%) patients walked less than the predicted distance. Their mean CTSS was notably higher at 8.5 ± 7, and they had a higher prevalence of reticular changes 15 (78.9%), along with a restrictive lung function observed in 11 (57.8%) patients, in comparison to the overall group.

Furthermore, 36 (35.6%) patients exhibited an HRR of 13 beats/minute or less. Within this subgroup, the majority, 31 (86%), had abnormal CT chest findings, characterized by either reticular changes 21 (58.3%) or GGO 10 (27.7%). Fibrotic changes were detected in 25 cases, predominantly featuring a mixed pattern encompassing subpleural lines, fibrous cord shadows, and traction bronchiectasis in 13 patients. However, no significant differences in the mean CTSS were noted based on HRR.

## UNIVARIATE ANALYSIS RESULTS

In our univariate analysis of radiological findings to PFT and 6MWT, we observed significantly lower prebronchodilator FVC (% of predicted), 92% vs. 81.1%, *p* = 0.01 as well as TLC (% of predicted), 94.3% vs. 82.5%, *p* = 0.02 (Table 5).

In our analysis of categorized CTSS, we found that patients with more severe CT abnormalities (CTSS scores in the range of 7–18) exhibited significantly lower D_LCO_, RV, and predicted walk distance, with *p* values of 0.01, 0.02, and 0.02, respectively. However, we did not identify any significant associations between radiological findings and other parameters in the 6MWT and PFT.

## DISCUSSION

In our study, we observed a significant improvement in chest CT findings among patients admitted to the ICU with COVID-19. However, even after 1 year following discharge from the ICU, a substantial 87% of these patients still exhibited abnormal chest CT scans. This aligns with the findings of many studies reporting longitudinal follow-ups of 1 year in COVID-19 patients, where residual radiographic abnormalities were documented in a range spanning from 24% to 87%.^3,5,17,24–28^

In cohorts representing similar severity of COVID-19 to our cohort, like Huang et al.’s study, encompassing patients requiring HFNC, NIV, or IMV, 87% exhibited at least one abnormal pattern in CT chest scans at the 1-year point. Notably, this cohort displayed a significant increase in subpleural lines (from 8% to 21%) and interlobular septal thickening (from 0% to 11%) between the 6-month and 1-year follow-ups. However, ground-glass changes decreased from 82% to 76% over the same period. Luger and colleagues documented extensive CT changes, including GGO, reticulation, bronchial dilation, and microcystic changes, in up to 55% of patients.^26,27^ In a systematic review, it was reported that patients with severe or critical COVID-19 had a higher prevalence of fibrotic-like changes, bronchiectasis, and interlobular septal thickening, in addition to GGO, which aligns with our cohort’s findings.^5^ In contrast, Bocchino et al.’s study focusing on patients with moderate COVID-19, who required oxygen support through nasal cannula and NIV but did not progress to ARDS, found that 93% of the 84 participants displayed normal CT chest results at the 1-year mark. The remainder exhibited fibrotic or fibrotic-like abnormalities.^3^ Vijayakumar et al., in a recent study involving 80 participants, where 40% of the individuals required NIV or mechanical ventilatory support, discovered that patients necessitating ventilatory support showed a higher rate of abnormal CT scans. Furthermore, only 16% of the 33 participants with abnormal CT scans at the 3-month mark displayed normal CT chest results at the 1-year follow-up.^24^

Our study reported a higher prevalence of residual abnormalities, with 68% exhibiting fibrotic changes and 66% displaying reticular changes at 1 year. This signifies a shift from the dominant mixed pattern of GGO with consolidation observed during acute severe COVID-19. Our study showed higher rates of residual CT abnormalities likely due to our cohort comprising severe and critical COVID-19. COVID-19 and CT abnormalities are prevalent more in those with critical COVID-19 and those with severe pneumonia and/or ARDS.^17,28^ Fibrosis in patients with COVID-19 has been secondary to pneumonitis itself and in ARDS secondary to the natural course of the disease or secondary to barotrauma in mechanical ventilation and its duration, hyperoxia, and a dysregulated immune response.^5,29^

It is important to highlight that a quarter of our patients who underwent a CT chest did not undergo PFTs. Among those who did complete PFTs, 47% exhibited abnormal lung function characterized by restriction. However, the mean spirometry, lung volume, and lung diffusion values all fell within normal limits. Notably, low D_LCO_ (<80%) was the predominant abnormality (50%) reported in pulmonary function at 1 year, which is consistent with findings from other studies involving patients with severe COVID-19, where this abnormality was observed in up to 30%–54% of cases.^5,26^ It is worth noting that despite differences in age and length of ICU stay between our cohort and a Chinese cohort from a similar study, there were no significant variations in D_LCO_ impairment.^25^ Furthermore, patients with a higher CTSS displayed a lower percentage of predicted D_LCO_ and RV. Those with abnormal CT chest findings also exhibited lower FVC and TLC, although the predicted values remained above 80%. Interestingly, at the 1-year mark, there was no significant correlation between lung function and radiological abnormalities.^24^

Regarding the 6MWT, our study found that 62% of patients walked less than the predicted distance, while only 18.8% experienced significant desaturation. Those who walked less than the predicted distance likely did so because of the exertional dyspnea as depicted by the modified Borg dyspnea scale moving from scale 1 prior to exercise in 72% of patients to scale 2 (41.5%), scale 3 (3.7%) and scale 4 (0.7%). This is also reported in 30%–35% of patients with severe COVID-19 at 1 year.^26,30^ Notably, a higher number of patients exhibited poor walk distance despite relatively few experiencing significant desaturation. This, along with the poor correlation with PFTs and radiological changes, suggests that functional impairment in these patients likely stems from factors beyond structural lung parenchymal abnormalities. This includes considering factors such as mitochondrial dysfunction with neuromuscular involvement, deconditioning, and long COVID symptoms that might explain the functional impairments despite normal or only mildly abnormal structural findings. Additionally, the interplay between physical deconditioning and psychological factors, such as anxiety and depression, may also contribute to the observed limitations in the 6MWT performance. These multifactorial contributors can further complicate the relationship between structural changes seen on imaging and functional outcomes assessed through PFTs and 6MWT. Therefore, it is crucial to adopt a comprehensive approach to evaluating and managing patients post-COVID-19, one that addresses not only the respiratory aspects but also the broader implications of long COVID and its associated symptoms. Such an approach may include rehabilitation strategies that focus on improving physical fitness, enhancing respiratory muscle strength, and addressing any psychological distress.^14,30–32^

The primary strength of our study lies in its random selection of all adult patients with severe COVID-19 who required ICU admission. These patients received uniform treatment and followed standardized infectious disease and ICU treatment protocols, which helps mitigate the bias associated with treatment heterogeneity. Despite demographic variations and lower COVID-19 mortality in Qatar, our overall findings align with those of other international studies. However, our study does have limitations, such as focusing only on patients with severe COVID-19 during the first and second waves of the pandemic. Although subsequent waves were less severe, it is unlikely that the outcome would differ significantly for patients developing severe disease even with less virulent strains. Moreover, the absence of pre-COVID-19 CT scans limits our ability to confirm whether minor interstitial abnormalities were present prior to infection, complicating the establishment of a baseline for evaluating radiographic changes. This necessitates a cautious interpretation of our findings regarding long-term abnormalities. While our study documents persistent structural changes post-COVID-19, the lack of baseline imaging means we cannot definitively attribute all observed abnormalities to post-infection sequelae; some may have been preexisting or reflective of the natural course of interstitial lung diseases. To address this, we have excluded patients with the following conditions from the study: moderate-to-severe COPD, bronchial asthma, cystic fibrosis, and parenchymal lung diseases, including interstitial lung disease. However, this exclusion does not entirely mitigate the limitation regarding the absence of baseline CT scans, as it does not account for other potential underlying conditions that may have influenced the radiographic findings. Although 135 out of the 204 enrolled patients completed follow-up CT scans and functional tests, the random dropout of participants may limit the completeness of the data. While this dropout was not systematic and does not introduce selection bias, it is acknowledged as a limitation that may impact the generalizability of our findings. Lastly, we acknowledge that potential treatment biases could influence outcomes, particularly given the variability in management approaches during the pandemic. Differences in treatment protocols—such as variations in the use of corticosteroids, antiviral agents, and supportive therapies—may contribute to discrepancies in recovery and long-term outcomes among patients. These variations, often dictated by evolving clinical guidelines and individual patient responses, highlight the complexity of treating severe COVID-19 and may impact the generalizability of our results. Additionally, the influence of healthcare resource availability during different waves of the pandemic could further affect treatment consistency and patient outcomes. Therefore, while our findings provide valuable insights, they should be interpreted with caution in light of these potential treatment biases and their implications for understanding the long-term consequences of COVID-19.

In conclusion, a significant proportion of patients with severe or critical COVID-19, followed up for 1 year, continued to exhibit one or more abnormal chest CT patterns. Reticular abnormalities were the predominant finding. Further studies are needed to explore interventions and therapeutic options, such as ventilatory strategies and antifibrotics, to mitigate structural lung changes and reduce the global burden of chronic lung disease following severe COVID-19. Despite relatively normal lung function parameters, our study revealed that the 6-minute walk distance was significantly impaired, likely due to neuromuscular abnormalities rather than isolated lung parenchymal abnormalities. Further research into the mechanisms behind significant functional impairment and therapeutic options is urgently required. In the interim, it is crucial to establish a structured follow-up plan for all severe COVID-19 patients, encompassing comprehensive evaluations and a thorough rehabilitation plan to enhance their exercise capacity and functional status.

## Conflicts of interest

This manuscript is an original one. It has not been published or considered for publication elsewhere. None of the authors have any financial or otherwise conflicts of interest from publishing this manuscript. The manuscript has been seen and agreed upon by all authors.

## Funding

This research was funded by the Medical Research Centre, HMC. The publication of this article will be funded by the Qatar National Library.

## Figures and Tables

**Table 1.** Demographic and clinical characteristics of patients with CT chest at 1-year follow-up.

**Baseline variables**	**All patients (*n* = 135)**	**Normal CT (*n* = 17)**	**Abnormal CT (*n* = 118)**	***p* value**
Age, years	48.8 ± 8.7	47.2 ± 9.8	48.9 ± 8.5	0.43
Male, *n* (%)	106 (78.5)	11 (64.7)	95 (80.5)	0.13
Female, *n* (%)	29 (21.5)	6 (35.2)	23 (19.5)	0.13
**Nationality**				
Non-Qatari, *n* (%)	126 (93.3)	15 (88.2)	111 (94.1)	0.36
Qatari, *n* (%)	9 (6.7)	2 (11.8)	7 (5.9)	0.36
**Comorbidities**				
Smoker, *n* (%)	9 (6.7)	0 (0)	9 (7.6)	0.23
Diabetic mellitus, *n* (%)	68 (50.4)	8 (47.1)	60 (50.8)	0.77
Hypertension, *n* (%)	65 (48.1)	7 (41.2)	58 (49.2)	0.53
Chronic cardiac disease, *n* (%)	6 (4.4)	0 (0)	6 (5.1)	0.34
Chronic kidney disease, *n* (%)	4 (3.0)	0 (0)	4 (3.4)	0.44
Chronic lung disease, *n* (%)	10 (7.4)	0 (0)	10 (8.5)	0.21
Cancer, *n* (%)	2 (1.5)	0 (0)	2 (1.7)	0.58
Pregnancy, *n* (%)	2 (1.5)	2 (11.8)	0 (0)	0.001
Immunocompromised, *n* (%)	3 (2.2)	0 (0)	3 (2.5)	0.50

**Table 2.** Biochemical, oxygenation: ventilation and prognostic characteristics of the patients.

**Baseline variables**	**All patients (*n* = 135)**	**Normal CT (*n* = 17)**	**Abnormal CT (*n* = 118)**	***p* value**
White blood cell count, ×10^3^/μL	12.2 ± 6.6	11.99 ± 5.2	12.2 ± 6.9	0.88
Absolute neutrophil count, ×10^3^/μL	10.0 ± 5.3	10.1 ± 4.9	9.98 ±5.5	0.93
Lymphocytes, ×10^3^/μL	1.04 ± 1.3	1.12 ± 1.4	1.03 ± 1.3	0.80
C-reactive protein, mg/L	149.3 ± 95.7	106.4 ± 92.33	154.3 ± 95.7	0.05
Procalcitonin, ng/mL	0.46 ± 2.5	0.05 ± 0.1	0.52 ± 2.7	0.48
Ferritin, /L	2597.2 ± 6508.9	1081.8 ± 1258.8	2802.7 ± 6899.2	0.32
D-dimer, mg/L	6.1 ± 13.4	3.52 ± 6.2	6.53 ± 14.2	0.39
Lactate level at day 1, mmol/L	1.4 ± 0.7	1.51 ± 0.6	1.45 ± 0.8	0.80
Lowest PaO_2_* to FiO_2_* ratio at day 1, *n* (%)				
Normal (≥400)	1 (0.8)	1 (6.3)	0 (0)	0.001
Respiratory failure (<300)	4 (3.1)	0 (0)	4 (3.5)	0.001
Severe respiratory failure (<250)	6 (4.6)	3 (18.8)	2 (2.6)	0.001
Critical respiratory failure (<200)	119 (91.5)	12 (75)	107 (93.9)	0.001
Duration of hypoxia, hours (PaO_2_ < 65 mmHg)	27.4 ± 48.2	8.6 ± 14.3	30.1 ± 5	0.001
**Assisted ventilation support, *n* (%)**				
IMV	32 (23.7)	3 (17.6)	29 (24.6)	0.53
Prone positioning	39 (28.9)	4 (23.5)	35 (29.7)	0.60
NIV	97 (71.9)	14 (82.4)	83 (70.3)	0.30
HFNC	98 (72.9)	10 (58.8)	88 (74.5)	
**Mechanical ventilation parameters (mean ± SD)**				
Tidal volume, mL	366.7 ± 106.3	460	361.5 ± 106.9	0.38
Compliance, mL/cm of H_2_O	25.1 ± 7.3	30.0	24.3 ± 7.1	0.52
**Medications, *n* (%)**				
Corticosteroids	134 (99.3)	17 (100)	117 (99.2)	0.70
Methylprednisolone	45 (33.3)	3 (17.6)	42 (35.6)	0.14
Dexamethasone	124 (91.9)	16 (94.1)	108 (91.5)	0.71
Remdesivir	89 (65.9)	9 (52.9)	80 (67.8)	0.22
Tocilizumab	33 (24.4)	2 (11.8)	31 (26.3)	0.19
Muscle relaxants	131 (97)	17 (100)	114 (96.6)	0.44
Sedative	31 (23)	3 (17.6)	28 (23.7)	0.57
Vasopressors	27 (20)	2 (11.8)	25 (21.2)	0.36
Convalescent plasma therapy	55 (40.7)	5 (29.4)	50 (42.4)	0.30
**Other variables**				
APACHE II death risk	10.45 ± 5.18	9.68 ± 10.57	8.48 ± 4.56	0.51
Delirium in ICU, *n* (%)	21 (15.6)	2 (11.8)	19 (16.1)	0.64
Length of stay in ICU, days, (mean ± SD)	10.8 ± 9.5	7 ± 3.6	11.3 ± 9.9	0.07
Total hospital stay, days, (mean ± SD)	19.9 ± 12.8	15.47 ± 5.95	20.64 ± 13.4	0.009

*PaO_2_ (arterial oxygen partial pressure obtained from an arterial blood gas) to the FiO_2_ (fraction of inspired oxygen).

**Table 3.** Chest CT features during admission with COVID-19 and at 1-year follow-up.

**CT chest finding**	**Patients at 1 year (*n* = 135)**	**Patients at baseline (*n* = 43)**	***p* value**
Total CTSS score (mean ± SD)	8.1 ± 6.8	19.4 ± 3.6	0.001
Abnormal CT, *n* (%)	118 (87.4)	43 (100)	
**Description, *n* (%)**						
Reticular	90 (66.7)	1 (2.3)	0.001
Pure GGO	48 (35.6)	2 (4.7)	0.004
GGO with consolidation	2 (1.5)	30 (69.8)	0.001
Mixed pattern*	30 (22.2)	10 (23.2)	0.77
**Fibrotic changes, *n* (%)**	92 (68.1)	10 (23.2)	0.001
Subpleural line	16 (11.9)	3 (6.9)	
Fibrous cord shadow	14 (10.4)	0	
Traction bronchiectasis	6 (4.4)	0	
Mixed pattern of fibrotic changes	56 (41.5)	7 (16.2)	

*Mixed pattern—Reticular/GGO/Consolidation.

**Table 4.** Lung function test and 6MWT at 1-year follow-up.

**Lung function parameter**	**Patients (*n* = 99)**
FEV1 (% of predicted), mean ± SD	
Prebronchodilator	84.3 ± 14.4
Postbronchodilator	84.7 ± 14.6
FEV1 <80%, *n* (%)	45 (45.5)
FVC (% of predicted), mean ± SD	
Prebronchodilator	82.4 ± 14.3
Postbronchodilator	81.1 ± 14.9
FVC <80%, *n* (%)	41 (41.4)
FEV1/FVC (%), mean ± SD	
Prebronchodilator	84.1 ± 5.8
Postbronchodilator	86.4± 4.47
PEF (% of predicted), mean ± SD	
Prebronchodilator	114.9 ± 19.6
Postbronchodilator	115.3 ± 18.8
TLC (% of predicted), mean ± SD	83.6 ± 14.17
D_LCO_ (% of predicted), mean ± SD	79.7 ± 16.4
D_LCO_ < 80%, *n* (%)	50 (50.5)
D_LCO_/V_A_ (% of predicted), mean ± SD	96.1 ± 14.2
RV (% of predicted), mean ± SD	91.5 ± 25.5
Abnormal spirometry^#^	47 (47.4)
Restrictive defect FEV1/FVC ≥70, *n* (%)	45 (45.4)
Obstructive defect FEV1/FVC <70, *n* (%)	2 (2.0)
**6 months walk test**	**Patients (n = 101)**
Walked distance (meters), mean ± SD	430.6 ± 50.4
Walked distance < predicted distance *n* (%)	63 (62.3)
**Oxygen saturation**	
**4% or more SpO_2_ decline, *n* (%)**	19 (18.8)*
CTSS	8.5 ± 7
SpO_2_ < 88% at the end of the test	0 (0)
Walked less than predicted distance	6 (31.5)
Reticular changes in CT chest	15 (78.9)
GGO in CT chest	3 (15.8)
Restrictive lung function	11 (57.8)
**HRR ≤13 beats/minute (HRR1), *n* (%)**	36 (35.6)*
Reticular changes in CT chest	24 (66.7)
GGO in CT chest	11 (30.6).
Fibrotic changes in CT chest	25
Mixed pattern of subpleural line, fibrous cord shadow, and traction bronchiectasis	8 (22.2)
Dyspnea on modified Borg scale** before exercise, *n* (%)	
Very slight, n (%)	98 (72.6)
Slight, n (%)	3 (2.2)
Dyspnea on modified Borg scale** after exercise, *n* (%)
Very slight	39 (28.9)
Slight	56 (41.5)
Moderate	5 (3.7)
Somewhat severe	1 (0.7)
Fatigue on modified Borg scale** before exercise, *n* (%)	
Very slight	98 (72.6)
Slight	3 (2.2)
Fatigue on modified Borg scale** after exercise, *n* (%)	
Very slight	84 (62.2)
Slight	16 (11.9)
Moderate	1 (0.7)

^#^Abnormal spirometry: FEV1 <80% or FVC <80%.

*P value: nonsignificant when compared to those with HHR1 >13 beats/minute or less than 4% SpO_2_ decline.

**modified Borg scale for dyspnea and fatigue: 0 Nothing at all, 0.5 Very, very slight (just noticeable), 1 Very slight, 2 Slight (light), 3 Moderate, 4 Somewhat severe, 5 Severe (heavy), 6,7 Very severe, 8,9,10 Very, very severe (maximal).

**Table 5.** Associations of abnormal CT chest findings at follow-up with pulmonary outcomes.

**Clinical parameter**	**CT radiology**			***p* value**
	**Normal**	**Abnormal**		
**Lung function test**			
FEV1 (% of predicted), mean ± SD			
Prebronchodilator	91.5 ± 14.6	83.3 ± 14.2		0.07
Postbronchodilator	91.5 ± 17.4	83.9 ± 14.2		0.14
FVC (% of predicted), mean ± SD						
Prebronchodilator	91.9 ± 15.4	81.1 ± 13.7		0.01
Postbronchodilator	89.1 ± 17.7	80.2 ± 14.4		0.090
FEV1/FVC (%), mean ± SD						
Prebronchodilator	82.9 ± 3.4	84.3 ± 6.0		0.44
Postbronchodilator	85.4 ± 3.5	86.6 ± 4.5		0.471
PEF (% of predicted), mean ± SD						
Prebronchodilator	112.8±19.21	115.3±19.8		0.69
Postbronchodilator	114.9 ± 14.2	115.4 ± 19.4		0.94
D_LCO_ (% of predicted)	87.7 ± 10.6	78.9 ± 16.8		0.13
D_LCO_/V_A_ (% of predicted)	94.3 ± 14.7	96.3 ± 14.3		0.70
TLC (% of predicted)	94.3 ± 12.7	82.5 ± 13.9		0.02
RV (% of predicted)	101.7 ± 21.4	90.6 ± 25.9		0.28
**6MWT**			
Heart rate at 6 minutes, beats/minute	108.8 ± 28.0	102.4 ±16.6		0.26
HRR 1 minute, beats/minute	19.2 ± 21.8	18 ± 13.5		0.78
O_2_ saturation at 6 minutes	98.3 ± .651	97.5 ± 1.2		0.033
Distance, meters, mean ± SD	434.4 ± 39.3	430.1 ± 51.9		0.78
Distance less than predicted, *n* (%)	7 (77.8)	63 (62.4)		0.02
**Associations of CTSSS**	**CTSS**			
	<7	7–18	>18	
D_LCO_ <70% of predicted, *n* (%)	5 (11.1)	14 (37.8)	3 (37.5)	0.01
RV <70% of predicted, *n* (%)	3 (7.1)	9 (28.1)	3 (42.9)	0.02
Distance less than predicted, *n* (%)	25 (49)	31 (75.6)	7 (11.1)	0.02
HRR ≤13 beats/minute, *n* (%)	20 (39.2)	14 (34.1)	2 (22.2)	0.59
